# A Traditional Costa Rican Adolescents' Diet Score Is a Valid Tool to Capture Diet Quality and Identify Sociodemographic Groups With Suboptimal Diet

**DOI:** 10.3389/fpubh.2021.708956

**Published:** 2021-08-12

**Authors:** Rafael Monge-Rojas, June O'Neill, Michelle Lee-Bravatti, Josiemer Mattei

**Affiliations:** ^1^Nutrition and Health Unit, Costa Rican Institute for Research and Education on Nutrition and Health (INCIENSA), Ministry of Health, Tres Ríos, Costa Rica; ^2^Department of Nutrition, Harvard TH Chan School of Public Health, Boston, MA, United States

**Keywords:** adolescents, traditional diet score, diet quality, socioeconomic status, Latin America

## Abstract

Traditional diet indices may capture diet quality according to local food culture. Higher adherence to traditional diet scores may help prevent disease, yet evidence in adolescents is limited. This cross-sectional study aimed to develop and validate a Traditional Costa Rica Adolescents Diet Score (TCRAD) and determine its association with sociodemographic characteristics, under the hypothesis that girls, adolescents from rural areas, and with low socioeconomic status, have a more traditional healthy diet. A total of 804 urban and rural adolescents (13–18 years old) participated in the study. The TCRAD showed adequate internal validity as shown by significant associations with intake of 14 traditional foods and nutrients (legumes, vegetables, fruits, oils, dairy, and corn tortilla scored as healthy; and white rice, red/processed meat, solid fats, desserts/pastries, sugar-sweetened beverages, snacks, fast food, and bread and cookies scored as unhealthy). A high TCRAD score, indicative of a healthier and more traditional diet, was observed among adolescents in the low socioeconomic group vs. medium or high socioeconomic categories (42.9, 41.2, and 38.2%, respectively, *p* < 0.05), adolescents living in rural areas vs. urban (47.6 vs. 34.2%, *p* < 0.05), and among boys vs. girls (46.9 vs. 37.5%, *p* < 0.05). The TCRAD score is a valid tool to capture diet quality of adolescents in Costa Rica and could be used to measure association of diet with disease outcomes in this and similar populations. Public health nutrition programs in Costa Rica should focus on improving intake of foods and nutrients, and prioritize girls, adolescents in urban areas, and adolescents with high socioeconomic status.

## Introduction

During the last 20 years, the diet quality of Costa Rican adolescents has deteriorated. Compared to adolescent dietary intake in 1996, adolescents in 2017 consumed fewer dairy products, vegetables, beans, and dietary fiber and more sugary drinks, pastries, desserts, snacks, fast foods, and total added sugars (1). Studies show that consuming a high-quality diet is associated with significant reductions in the risk of major chronic diseases (2, 3), suggesting that current dietary trends in Costa Rica may place adolescents at a high risk for these conditions. Furthermore, evidence suggests that healthy dietary habits established during adolescence continue into adulthood (3–6); hence, adolescence has been identified as the best time to achieve dietary modifications that seek to enhance health-conscious dietary habits (3–7). However, challenges remain in developing successful healthy eating strategies for adolescents, and moreover, inaccurately assessing diet quality in this age group (8, 9).

Traditional diet scores have been developed in Mediterranean countries (10), Japan (11), Mexico (12, 13), and Sweden (14) to assess and guide an individual's dietary intake that align with cultural preferences. The use of international population-specific diet quality scores has been recommended because their components reflect staple traditional food groups (15). When used in diet-disease epidemiological studies, these scores have been shown to be associated with lower risk of chronic diseases, often more strongly than diet quality scores developed in a different population (16–18). Using diet quality scores based on the dietary patterns of one population group to evaluate the diet of another one may not be adequate because some foods or nutrients included in the score may be under or over-represented in the new population under study (15). One solution is to use culturally appropriate food and nutrient components to define a traditional diet quality score adapted to the specific food culture of a population group, as it has been done in Mexico, Japan and Sweden (11–14).

Several studies have shown that sociodemographic factors and dietary habits are associated. It has been suggested that health inequalities can be partly explained by the dietary differences between various social backgrounds, such as area of residency and socioeconomic status (SES) (19, 20). A systematic review based on data from low- to middle-income countries showed variation in dietary patterns by national income or SES, indicating that SES plays an important role in diet quality variations (21). Various studies have found that BMI is generally higher in urban areas than in rural areas, which is driven by higher SES (22–26). Income growth enables households of high SES to access more food, both nutrient-dense foods that contributes to a high-quality diet as well as energy-dense, salty, or sugary food that can undermine diet quality (25, 26). Alternatively, other studies have indicated that the inhabitants of rural areas and low SES of middle income countries, maintain a traditional dietary pattern and have fewer resources to buy modern packaged processed foods that tend to be unhealthy (27–29).

Even so, few studies—particularly in Latin America—have analyzed the diets of adolescents living in rural vs. urban areas or belonging to different socioeconomic strata. These studies generally show that adolescents from rural areas tend to have a healthier diet, as we have shown in Costa Rica, a middle-income country (29, 30), although results from other countries have been mixed (31–34). Similarly, results on diet by SES are contradictory, although most point toward better diet with higher SES (35–37). In Costa Rica, foods associated with the nutrition transition have been shown to be mostly consumed by adults of higher SES (38). Lastly, sex has been identified as another determinant of diet quality (39, 40). There is consensus in the literature about girls having healthier dietary habits than boys; studies from Costa Rica generally support this (37, 41–43). Consumption of meat and high-energy dense foods (e.g., fast food, sugar-sweetened beverages) is higher among boys; meanwhile the consumption of vegetables, fruits, and other healthy foods is higher in girls (39).

Values assigned to food are shaped by the dynamic interaction between individuals and their socio-cultural environment over time (44). Therefore, understanding how diet quality varies between sociodemographic groups is vital for decision-makers to define sustainable improvement strategies based on the specific needs of each population subgroup. The purpose of this study was to develop and validate a Traditional Costa Rican Adolescents Diet (TCRAD) score and determine its sociodemographic correlates. Identifying sociodemographic characteristics correlated to the TCRAD can help prioritize subgroups at highest nutritional needs. We hypothesized that girls, adolescents from rural areas, and those of low socioeconomic status, have a more traditional healthy diet.

## Methods and Materials

### Study Population and Setting

This study used cross-sectional data from adolescents enrolled in rural and urban schools in the province of San José, Costa Rica, in 2017. San José has the highest adolescent concentration (30%) in the country (45). The majority of Costa Rican adolescents (80%) are enrolled in school (46). A total of 16 high schools were selected from a list of all the public and private high schools. Seventh to eleventh graders (13–18 years old) enrolled in the selected schools were invited to participate in the study.

The sample size of the study was determined assuming a sampling error for a population proportion using a 95% confidence interval and a permissible error of 5% and a finite population correction (47) Based on the last Costa Rican adolescent population census (45), the study sample was distributed to contemplate 53% girls and 46% boys.

The selection of the sample was carried out in a three-stage way. In the first stage, schools were selected using a proportional-size probability method (48). In the second stage, 10 classrooms (2 from each grade) were selected in each school using simple random sampling. In each section all students were invited to participate in the study. The informed assent form was explained to adolescents interested in participating in the study. If they agreed to participate, they were given an informed written consent form to take to their home and obtain their parents' permission to participate in the research study. In a third stage, the participants in the study were chosen randomly from among those students who returned signed informed consent and assent forms. Approximately to 5% of the initial sample chose not to participate in the study before it started. The final sample study was 818 adolescents aged 13–18 years.

## Study Variables

### Sociodemographic Variables

A paper-based questionnaire was used to collect data on sex, age, area of residence, parental education level, ownership of goods, and access to services (e.g., computers, internet, router, cable television, and water heating for the whole house), number of people in the household, and number of bathrooms in the house. To determine the socioeconomic status (SES), we applied the methodology proposed by the National Census and Statistics Institute (49). In brief, a score was determined using the sum of the points assigned to 12 variables categorized into different options. The total score ranged from 0 to 100 points. High SES was defined as a score ≥ 85 points, medium SES as a score between 30 and 84 points, and low SES as a score ≤ 29 points.

#### Dietary Intake Assessment

Dietary intake data were collected via 3-day food records completed by the participant in real time and reviewed by nutritionists. Participants were asked to complete a 3-day food record on 2 weekdays (Monday, Tuesday, Thursday, or Friday) and 9 weekend day (Saturday or Sunday). Half of the participants were randomly selected to record the foods and drinks that they consumed on Thursday, Friday, and Saturday, and the rest were asked to record their intake on Sunday, Monday, and Tuesday. Data were collected during 9 months of the school year (February–November), reflecting seasonal variations for Costa Rica: rainy season (May–November) and dry season (December–April). The goal was to ensure that the data captured daily and seasonal variability in food consumption.

At each school, six trained nutritionists provided participants with a notebook divided in three sections (one section per day), and each section subdivided by meal times (pre-breakfast, breakfast, between breakfast and lunch, lunch, between lunch and dinner, dinner, between dinner and bedtime). They were then taught how to complete accurate food records by writing detailed descriptions of what they ate and drank from the time they woke up in the morning to the time they went to bed at night, for three consecutive days. Adolescents were asked to write down brand names of foods when appropriate, methods of preparation, and recipes for all the dishes and drinks whenever possible. The nutritionists taught the participants how to estimate the portion sizes of the foods and drinks they consumed using an established portion-size manual developed for Costa Rica (50). The manual incorporates full-color photographs and diagrams of typical local foods and their preparation, including 3–6 different portion sizes. Each adolescent received the notebook and manual described above to estimate and record food portion sizes during 3 days. Adolescents were also instructed to report portion size using measurements based on household utensils or volume and mass units.

Given the challenges with incomplete and inaccurate data when recording self-reported dietary data in young populations and specific demographic groups (51), the completed 3-day food records were thoroughly reviewed by the nutritionists by conducting one-on-one reviews with each participant during school hours. At this interview, the nutritionists inquired about commonly missed items or ingredients (i.e., added sweeteners, added fats, candies, beverages); added details about the types of food or drinks that were consumed; verified or added any omitted portion sizes, and clarified any illegible items. The nutritionists used food models, fresh foods, and different utensils to verify serving and portion sizes.

The 3-day food records were used to estimate usual food consumption and to evaluate intra-individual variability in nutrient intakes. The web-based statistical modeling technique Multiple Source Method (MSM; https://msm.dife.de/tps/en), proposed by the European Prospective Investigation into Cancer and Nutrition (EPIC), was used to estimate energy and macronutrient intakes. This method was chosen because of its capability to improve estimates of usual dietary intake of energy and nutrients by considering within-person variance in intake, thereby improving the usual intake distribution for the population (52). Also, the method has been widely used in other Latin American studies to estimate usual intake (53, 54).

#### Development of the Traditional Costa Rican Adolescents Diet (TCRAD) Score

The TCRAD score, was developed by drawing from the components and scoring methodologies of the Mediterranean Diet Score (10), and adaptations made for the traditional Japanese, Mexican, Swedish, and Finnish diet scores (11–14, 55). Fourteen foods or nutrients groups were selected based on cultural preferences and sufficient consumption in the population (56) (Table 1). Six food groups were considered healthy based on the literature and previous indices (legumes, vegetables, fruits, oils, dairy, and corn tortilla), and eight food groups were considered unhealthy (white rice, red/processed meat, solid fats, desserts/ pastries, sugar sweetened beverages, snacks, fast food, and bread and cookies). While other diet quality indices include alcohol, whole grains, and nuts components, these were excluded from the TCRAD score because consumption among Costa Rican adolescents was negligible (5.98, 2.78, and 0.72 g/day, respectively). Sodium consumption was not included due to the lack of information on the sodium content of home-prepared meals in Costa Rica, which may lead to inaccurate estimates.

**Table 1:** Components and median cut-off values of the Traditional Costa Rican Adolescents Diet score.

**Food group**	**Examples of food items**	**Median cutoff for males (g/day)**	**Median cutoff for females (g/day)**
**Healthy foods**			
Legumes	Beans, lentils, chickpeas	42.9	25.6
Vegetables	All fresh vegetables (e.g., tomato, carrot, cabbage, etc.) except potato	32.6	41.6
Fruits	All fruits (e.g., orange, pineapple, banana, mango, etc.) except fruit juices	48.0	57.2
Vegetable oils	Soybean oil, corn oil, sunflower oil, etc.	7.4	5.6
Dairy	“Queso fresco” (soft cheese), milk, yogurt.	69.2	68.9
Tortillas	Corn tortilla	0.9	0.3
**Unhealthy foods**			
White rice	White rice	165.7	116.0
Red/processed meat	Steak, ground beef, sausage, mortadella, pork ham, processed turkey, etc.	43.7	28.3
Solid Fats	Margarine, butter, cream cheese, sour cream, etc.	4.2	4.3
Desserts	Rice pudding, coconut flan, Chilean thousand layers cake, cakes, etc.	29.3	35.3
Sugary drinks	Commercial sugar-sweetened beverages, beverages with added sugar prepared at school and at home	308.4	230.10
Snacks	Fried cassava/plantain or tortilla chips, salty/sweet extruded corn snacks, salty/sweet puffed corn snacks	8.9	11.4
Fast food	Empanadas (deep-fried corn dough turnover filled with meat, chopped potato, mashed beans or farmer's cheese), hamburgers, French fries, fried chicken	23.7	14.1
Refined bread	Baguette, white bread roll, homemade bread, sweet bread, sliced bread, cookies	47.4	43.3

*For healthy foods, a score of 0 points was assigned to individuals with intake (g/day) less than the sex-specific median, and 1 point to those for meeting or exceeding the sex-specific median cutoff. Inverse scoring was applied for unhealthy foods. All the scores of the components were summed together to create the total index score with a possible range of 0–14*.

Healthfulness allocation was based on previous scores, which account for nutrient content and evidence of health prevention or disease risk. Specifically, legumes, vegetables, fruits, oils, and red/processed meat were classified following the Mediterranean Diet Score (10). Corn tortillas, solid fats, dairy, and refined foods (e.g., desserts/ pastries, and breads and cookies) were scored following a traditional Mexican diet score (12, 13). Sugar sweetened beverages are characterized by a high content of added sugars, and snacks and fast foods are generally ultra-processed and energy-dense, which are detrimental to health and scored negatively in previous indices (55). Lastly, we included white rice as a separate food group given its large consumption as a staple food in Costa Rica with evidence of adverse health outcomes in this and other populations (57, 58).

For healthy foods, a score of 0 points was assigned to individuals with intake (grams/day) less than the sex-specific median, and 1 point to those for meeting or exceeding the sex-specific median cutoff. Inverse scoring was applied for unhealthy foods, similar to the Mediterranean diet score (10).

The scores of all components were summed together to create the total index score with a possible range of 0–14 highest TCRAD score corresponds to closer adherence to a more-traditional Costa Rican adolescent dietary pattern. The continuous score was categorized as high score (range: 8–14), moderate score (range: 6–7), low score (score <6), based on the inter-quartile range.

### Statistical Analysis

Epi Info™ software, version 3.5.4 (2008), was used to process the data from the 3-day food records. The food composition database used with this tool contains 1,655 items from the following sources: 1,307 foods from the US Department of Agriculture, 80 foods from the food composition tables of the Institute of Nutrition for Central America and Panama (INCAP), 254 foods from recipes commonly consumed in Costa Rica, and 13 foods from the Costa Rican mandatory fortification food group.

Fourteen participants were excluded from the analytic sample study because they reported energy intakes regarded as outliers. In dietary intake data, outliers can be the result of misreporting or coding errors, or they may reflect accurate self-report of non-normative food consumption. Outliers may not be representative of the population's normal intake distribution and can introduce biases and adversely impact statistical analysis and inferences. We used sex-specific unweighted total energy intake in the 1st percentile or above the 99th percentile as thresholds for outliers, as recommended by Dyer et al. (59). As a result, 804 participants remained in the final analysis.

Mean intake was adjusted for total energy intake using the residuals method in a regression model with the nutrients as the dependent variable and energy as the independent variable (60). Although all nutrient residuals displayed normality or near normality when plotted, some distributions were skewed. These data did not conform more closely to the normal distribution by log transformation, therefore we chose to not log transform the data. To determine the differences between the general characteristics of the analytic sample study, Chi Square was used for categorical variables and Student's *t*-test for continuous variables.

Correlation between daily nutrient intake of participants and TCRAD scores were calculated. Statistical significance was determined from Spearman's Rank Order correlation. Energy adjusted median values of diet quality score components were compared across the three TCRAD scores. Pairwise multiple comparison analysis was conducted and statistical significance was determined from the standardized Wilcoxon statistic. To identify factors associated with the TCRAD score, we tested associations between the scores and sex, age, SES, school type (public/private), and residential area (rural/urban) using a non-parametric ANOVA test (Kruskal Wallis). SAS version 9.4 (SAS Institute) was used for all analyses. All tests were 2-tailed and the statistical significance level was set as 0.05.

## Results

The analytic sample study had significantly more girls than boys (64.3 vs. 36.1%, *p* < 0.001). The mean age was 15.2 ± 1.7 years. The prevalence of overweight was significantly higher in urban adolescents compared to their rural counterparts (27.4 vs. 20.3%, *p* < 0.05). The prevalence of obesity was significantly higher in adolescents of high SES compared to those of low SES (12.3 vs. 9.6%, *p* < 0.05) and in boys compared to girls (12.4 vs. 8.3%, *p* < 0.05).

Median intake of energy-adjusted 14 components of the TCRAD score across the CRHEI categories is shown in Table 2. For all six healthy components (beans, vegetables, fruits, vegetable oils, dairy, and tortilla), energy-adjusted median intakes were significantly lower for adolescents with low TCRAD score as compared to adolescents with moderate or high TCRAD score. For the seven unhealthy components (red meats, solid fats, desserts, sugary drinks, snacks, fast foods, and bread consumption), energy-adjusted median intakes were significantly lower for adolescents with high TCRAD score vs. low traditional score. However, while considered an unhealthy food and inversely scored, median intake of white rice was significantly higher in adolescent with high TCRAD score vs. low traditional score.

**Table 2:** Median (min-max) of energy-adjusted components intakes of the overall and by categories of the Traditional Costa Rican Adolescents Diet (TCRAD) score (*n* = 804)^1^.

**TCRAD components^**1**^**	**TCRAD score range (0–13)**				***P*-value^**3**^**
	**Overall**	**Low** ^**2**^ **(Score <6)**	**Moderate** ^**2**^ **(Score = 6–7)**	**High** ^**2**^ **(Score 8–14)**	
***Components—positive scoring***	***n =* 804**	***n =* 133**	***n =* 344**	***n =* 327**	
Beans, g/d	32.4 (0.11–233.2)	22.6 (0.11–176.2)	31.1 (0.17–233.2)	50.3 (0.17–223.9)	<0.0001^‡^
Vegetables, g/d	44.4 (0.31–635.3)	26.8 (0.53–203.5)	42.4 (0.31–635.3)	50.5 (0.56–460.6)	<0.0001^‡^
Fruits, g/d	62.1 (0.27–776.5)	33 (0.85–589.5)	56.6 (0.76–624.6)	82 (0.27–776.5)	<0.0001^‡^
Vegetable oils, g/d	6.3 (0.05–24.1)	4.6 (0.09–15)	5.7 (0.11–24.1)	7.6 (0.05–20.9)	<0.001^‡^
Dairy, g/d	94.8 (0.03–1536.3)	63.2 (0.4–828.7)	94.8 (0.03–1020.6)	105.6 (0.4–1536.3)	<0.001^a, b^
Tortillas, g/d	0.8 (0–170.2)	0.6 (0.01–170.2)	0.7 (0–54.2)	1.6 (0.02–93.3)	<0.05^a, b^
**Components—negative scoring**					
White rice, g/d	127.2 (0.14–566.6)	118.7 (7.55–337.8)	130.1 (0.14–469.2)	141.4 (0.98–566.6)	0.001^b^
Red meat, g/d	38.5 (0.01–252.4)	42.6 (2.83–176)	44.6 (0.01–252.4)	34.4 (0.26–200.8)	0.0047^b, c^
Solid Fats, g/d	5.6 (0.02–366.5)	6.5 (0.04–159.8)	6.2 (0.07–366.5)	4.7 (0.02–89.5)	<0.0001^b, c^
Desserts, g/d	37.6 (0.27–443.2)	45.2 (1.25–262.7)	38.4 (0.27–443.2)	29 (0.64–314.2)	<0.05^‡^
Sugary drinks, g/d	265.6 (0.79–2179.3)	368.2 (17.44–1484.9)	286.8 (1.05–2179.3)	211.1 (0.79–1953.8)	<0.01^‡^
Snacks, g/d	13.6 (0.01–152.4)	15.5 (0.03–89.1)	13.7 (0.01–152.4)	10.7 (0.09–102.7)	<0.0001^b, c^
Fast food, g/d	28.8 (0.01–441.5)	39.2 (0.18–441.5)	30.6 (0.01–402.6)	25.8 (0.3–310.8)	<0.001^b^
Refined bread, g/d	46 (0.04–256.5)	62.2 (2.12–249.8)	50.2 (0.04–256.5)	37.5 (0.42–214.2)	<0.005^‡^

*^1^Intake adjusted for energy. ^2^ Diet quality categories determined by interquartile range values of score distribution. ^3^P-values generated by Wilcoxon analyses comparing median values between diet quality categories. Pairwise comparisons significance (^a^Low score TCRAD diet-Average TCRAD, ^b^High score TCRAD, ^c^Average TCRAD), ‡significant across all pairwise comparisons*.

Overall, the TCRAD score was significantly correlated with all tested nutrients except total energy, sugar, cholesterol, and calcium (Table 3). Total fat, mono-, poly-, and saturated fats were inversely and weakly correlated with the TCRAD score. Fiber, folate, magnesium, and potassium showed moderate positive correlation with the TCRAD score.

**Table 3:** Daily total energy and nutrient intake of participants and correlation with the Traditional Costa Rican Adolescents Diet score^a^.

**Nutrients^**b**^**	**Daily intake**		**Spearman's Rank** **Order correlation** **(rho)**	**Interquartile ranges**
	**Median**	**Mean (SD)**		
Energy (kcal)	1906.5	1937.9 (466.1)	−0.01	1589.3–2259.1
Carbohydrate (g)	291.3	289.7 (29.3)	0.13***	291.3–307.9
Fiber (g)	14.7	15.2 (4.3)	0.38***	14.7–17.7
Sugar (g)	102.4	102.5 (27.8)	−0.05	102.4–120.5
Protein (g)	61.5	62.2 (9.6)	0.12**	61.5–68
Total fat (g)	59.9	60.2 (9.5)	−0.20***	59.9–66.1
Monounsaturated fats (g)	20.7	20.8 (3.5)	−0.14***	20.7–22.9
Polyunsaturated fats (g)	12.1	12.3 (2.4)	−0.08*	12.1–13.5
Saturated fats (g)	19.2	19.4 (4.2)	−0.23***	19.2–21.6
Cholesterol (mg)	203.4	209.8 (62.3)	0.05	203.4–243.8
Vitamin A (mcg)	214.3	220.0 (76.4)	0.11**	214.3–261.4
Vitamin B6 (mg)	1.5	1.5 (0.4)	0.17***	1.5–1.7
Vitamin B12 (mcg)	3.8	4 (1.2)	0.07*	3.8–4.5
Vitamin C (mg)	95.7	102.3 (42.5)	0.21***	95.7–123.5
Vitamin D (mg)	51.6	55.7 (28.2)	0.07*	51.6–70.8
Vitamin E (mg)	6.5	6.8 (2.2)	0.07*	6.5–7.7
Vitamin K (mg)	66.4	68.5 (23.1)	0.28***	66.4–81.6
Folate (μg)	489.7	498.3 (119.1)	0.34***	489.7–566.8
Calcium (mg)	570.3	583.9 (191.3)	0.04	570.3–687.7
Magnesium (mg)	230.0	232.3 (39.2)	0.39***	230–256.1
Iron (mg)	12.4	12.9 (2.9)	0.15***	12.4–14
Potassium (mg)	2072.1	2109.4 (387.6)	0.36***	2072.1–2336.4
Zinc (mg)	8.7	8.8 (1.5)	0.17***	8.7–9.7

^a^1st and 99th percentiles for energy intake were excluded, ^b^Nutrients adjusted for energy intake. P-values generated using Spearman correlations. Significant correlations between nutrients and diet quality scores shown as *p < 0.05, **p < 0.01, ***p < 0.0001.

Dietary intakes of TCRAD score components by adolescents' sociodemographic. characteristics are presented in Table 4. Mean energy-adjusted TCRAD score was higher in girls than in boys (7.5 vs. 7.0, *p* = 0.0004), lower in adolescents classified as high SES compared to those classified as medium and low SES (6.9 vs. 7.2 vs. 7.3, *p* < 0.05), and higher in adolescents living in rural than those living in urban areas (7.5 vs. 6.8, *p* < 0.0001). Significant differences were not detected across age groups.

**Table 4:** Mean intake of the Traditional Costa Rican Adolescents Diet (TCRAD) score components, by sociodemographic characteristics^a^.

	**Overall** **(** ***n*** **=** **804)**		**Sex** ^****b****^				**Area of residence** ^**b**^				**Socioeconomic status** ^****c****^						**Age group** ^****a****^			
			**Boys** **(** ***n=*** **293)**		**Girls** **(** ***n=*** **511)**		**Urban** **(** ***n=*** **403)**		**Rural** **(** ***n=*** **401)**		**Low** **(** ***n=**>* **259)**		**Medium** **(** ***n=*** **319)**		**High** **(** ***n=*** **226)**		**12–15 y** **(** ***n=*** **470)**		**16–19 y** **(** ***n=*** **334)**	
	**Mean**	**SD**	**Mean**	**SD**	**Mean**	**SD**	**Mean**	**SD**	**Mean**	**SD**	**Mean**	**SD**	**Mean**	**SD**	**Mean**	**SD**	**Mean**	**SD**	**Mean**	**SD**
TCRAD^d^	7.2	0.2	7.0	0.1	7.5	0.1**	6.8	0.1	7.5	0.1	7.3	0.1	7.2	0.1	6.9	0.1	7.2	0.1	7.1	0.1
Dairy^e^, g/d	132.8	135	149.0	156	123.4	120*	146.8	143	118.6	125**	106.1	95	139.6	147	153.7	151**	139.2	135	123.7	135
Red meat, g/d	47.3	36	55.7	36	42.4	35***	49.4	39	45.2	33	46.9	39	45.3	32	50.7	38	48.2	36	46.2	36
White rice, g/d	147.3	94	183.9	108	126.2	77***	125.2	83	169.6	99***	185.7	103	145.3	87	106.2	71***	147.4	93	147.2	94
Beans, g/d	48.2	43	60.7	49	40.9	35***	39.8	38	56.6	45***	59.7	49	47.7	40	35.6	32***	47.6	42	48.9	43
Corn Tortilla, g/d	6.8	14	8.4	15	5.9	12*	6.6	14	7.0	13	5.7	11	7.5	14	7	15	7.2	15	6.2	11
Vegetables^f^, g/d	61.1	63	57.7	64	63.0	60	48.8	56	73.5	65***	63.9	59	62.9	65	55.3	61	58.1	60	65.3	64
Fruits, g/d	90.3	96	90.7	109	90.1	86	86.2	97	94.5	94	99.3	103	84.4	83	88.4	102	86.9	94	95.2	97
Vegetable oils^g^, g/d	6.8	4	8.03	3	6.14	3***	6	3	7.7	4***	7.8	4	6.6	3	6.1	4***	6.8	4	6.8	6.8
Solid fats, g/d	11.1	21	13.8	29	9.58	13*	13.4	27	8.8	12**	10.9	25	11	17	11.5	21	11.6	24	10.5	17
Desserts, g/d	47.9	42	47.1	42	48.3	41	45.4	37	50.3	46	46.3	40	48.2	46	49.3	37	45.8	42	50.8	41
Sugary drinks^h^ g/d	324.8	253	382.5	297	291.7	217***	372.5	276	276.6	218***	303.2	226	326	234	347.9	303***	327	241	321.7	269
Refined bread, g/d	51.7	35	58.6	40	47.7	31***	56.6	37	46.8	33***	46.4	33	55.3	38	52.6	34**	49.2	34	55.2	38*
Snacks^i^, g/d	17.3	16	15.5	14	18.3	16*	18.6	15	16.1	17*	17.4	15	17.2	15	17.5	18	17.4	16	17.3	16
Fast foods^j^, g/d	49.2	57	60.8	67	42.5	48***	54.0	59	44.4	54*	38.3	45	45.4	51	67.2	70***	51.4	57	46.1	55

^a^Values given as mean ± SD, adjusted for energy intake and gender. ^b^P-values were determined using Independent Samples t-Test, ^c^P-values were determined using ANOVA. Significant differences determined shown as *p < 0.05, **p < 0.01, ***p < 0.0001, as compared to the referent group (Boys, urban, low socioeconomic status, and or youngest age group), ^d^Mean energy-adjusted score, ^e^Includes milk, cheese, and yogurt, ^f^Does not include potatoes, ^g^Includes soybean oil, sunflower oil, corn oil and rapeseed (canola) oil, ^h^Includes industrialized sugar-sweetened beverages, drinks with added sugar prepared at school and at home, ^i^Includes fried cassava/plantain or tortilla chips, salty/sweet extruded corn snacks, salty/sweet puffed corn snacks, ^j^Includes empanadas (deep-fried corn dough turnover filled with meat, chopped potato, mashed beans or farmer's cheese); Costa Rican tacos (deep-fried tortilla filled with meat and topped with shredded cabbage and a generous amount of ketchup and mayonnaise); special croissants (croissant filled with bologna, beef, or processed American cheese and fresh tomato); arreglados (pastry shells filled with meat, mashed beans, and fresh tomato); wraps (soft flatbread rolled around a filling like cold sliced meat, poultry or fish, vegetables and a sauce); hot dogs, hamburgers, nachos, pizza, sandwiches, burritos, French fries, and fried chicken, among others.

Compared to girls, boys reported significantly higher intakes of dairy products, white rice, tortillas, vegetable oils, sugary drinks, bread, snacks, and fast foods. Adolescents in rural areas consumed significantly more white rice, beans, and vegetables compared to urban residents. In contrast, adolescents in rural (vs. urban) areas reported significantly lower intakes of dairy products, vegetable oils, solid fats, sugary drinks, bread, snacks, and fast foods. Compared with adolescents classified as low SES, those classified as high SES consumed significantly more dairy products, sugary drinks, bread, and fast foods. In contrast, adolescents of high SES consumed significantly less white rice, beans, and vegetable oils. There were no differences in TCRAD components by age category.

Compared to “moderate” and “low” TCRAD score, most boys were considered to have a “high score” (46.9%), while most girls maintained a “moderate score” (43.5%) (Supplementary Figure 1). Fewer boys (12.6%) were in the “low score” than girls (19.0%). More adolescents in the low SES group had high TCRAD score compared to those in the medium or high SES groups. Adolescents living in rural areas were more likely to have high score (47.6%) vs. their urban counterparts (34.2%). There were twice as many urban than rural-residing adolescents with high TCRAD score (22.0 vs. 11.4%).

## Discussion

Based on previously used definitions for traditional diet scores in other populations, we adapted a Traditional Costa Rican Adolescent Diet (TCRAD) score and demonstrated adequate internal validity. Using the TCRAD score revealed that only about 40% of Costa Rican adolescents had a closer adherence to a more-traditional Costa Rican adolescent dietary pattern. This is closely linked to intake of beans, vegetables, fruits, dairy products, and oils that score positively for the diet quality score and that are rich in diverse micronutrients. According to our hypothesis, both adolescents from rural areas and those of low socioeconomic status had a more traditional and healthy diet; however, we rejected the hypothesis for differences by sex, such that girls had significant lower traditional diet quality than boys.

Intake of fiber, folate, magnesium, and potassium was higher among adolescents with higher TCRAD score. Strategies designed to increase the consumption of these nutrients-rich foods (such as beans, fruits, and vegetables) as part of a healthy diet among adolescents should be implemented to prevent non-communicable chronic disease starting in young adulthood. These nutrients have multiple biological effects, including antioxidant and anti-inflammatory activity and angiogenesis, that may help explain their association with reduced risk for all-cause mortality, cardiovascular disease, cancer, and diabetes (61).

There was little variability in total sugar consumption, which may explain the absence of correlation between total sugar consumption and the TCRAD score. Given the association between high sugar consumption and the risk of obesity and cardio-metabolic disease (49), approaches to lower sugar intake and improve the adolescent diet are of high public health interest.

In contrast to the findings of other studies (62–66), a higher proportion of male adolescent had a high TCRAD score, despite higher intakes of foods that negatively contributed to it. Since boys and girls had similar intakes of fruits and vegetables, the higher diet quality in boys may be because of higher intake of beans and dairy products. The daily intake of these foods has been identified as part of the dietary pattern of long-lived people in the “Blue Zone” of the Nicoya Peninsula in Costa Rica (67).

Interestingly, urban adolescents and those of higher SES consumed more dairy products, but the TCRAD score was lower in this group compared to their rural and lower SES counterparts. Nonetheless, adolescents in urban areas and those of higher SES consumed more high-fat and high-sugar foods, which could reduce the positive role of dairy products on the TCRAD score.

A large body of epidemiologic data shows that diet quality follows a socioeconomic gradient. Higher educational attainment and SES are generally associated with high-quality diets, while groups of lower SES tend to consume poor-quality diets (64, 68–71). However, our results reveal an inverse relationship between the TCRAD score and SES.

A substantial proportion of Costa Rican adolescents living in rural communities, and those having a lower SES had a high TCRAD score. This result may be explained by their higher intake of beans, and lower intake of sugary drinks and energy-dense foods, such as snacks and fast food. Beans are high in fiber, folate, magnesium, and potassium (72), nutrients that had the highest positive correlations with the diet quality score. In contrast, the correlation between high-fat diets and the diet quality score was negative (rho = −0.20). The high TCRAD score observed in adolescents living in rural areas and with low SES suggests a closer adherence to the traditional diet, as noted in other rural populations (66, 73). Meanwhile, the low TCRAD score observed among those adolescents living in urban areas and with higher SES seems to reflect nutritional transition.

Nutritional transition is characterized by the progressive shift from a traditional diet rich in fiber and low in fat and sugar, to a diet rich in animal products, refined grains, fats, salt, and sugar, but low in fiber (74). This type of diet has been linked to a higher risk of obesity and nutrition-related chronic diseases (75). It has been thoroughly demonstrated that rapid urbanization and high-income households are critical drivers of this transition (76–79). The higher prevalence of overweight and obesity among urban adolescents and those of higher SES is consistent with those findings, which is quite alarming: out of five Central American countries, Costa Rica already has the lowest percentage (9%) of adults (20 years or older) that are free of any metabolic syndrome components (80). In addition, the consumption patterns of urban, high-income households are shifting as part of the nutritional transition, and additional income is commonly used to purchase more energy-dense foods (81, 82). A wide gap is emerging between population groups that can afford more expensive, highly processed foods and the more deprived groups that maintain their traditional diets (83).

Contrary to the detrimental effects on public health of disparities in the distribution of social, political, economic and environmental resources reported previously (84), the diet-disparities we observed may translate into a lower risk factor profile of chronic non-communicable diseases in Costa Rican adolescents living in rural areas and in those of low socioeconomic status.

Several epidemiological studies have shown that a higher intake of white rice is associated with an increased risk of type 2 diabetes (57, 58, 85, 86). However, our index showed that adolescents with high TCRAD score consumed plenty of white rice. This finding may seem contradictory; however, in the Costa Rican dietary pattern, white rice and beans are staple foods typically consumed together (87–89). Mattei et al. (88) observed that a higher ratio of beans to rice was associated with a 35% risk reduction of metabolic syndrome among Costa Rican adults. The combined intake of rice and beans may explain why there is a larger proportion of carbohydrates and proteins across high TCRAD score.

TCRAD score was designed by incorporating the most recent scientific evidence on the relationship between diet and health. While creating culturally-tailored diet scores based on a population's preferences and intake distributions may limit comparison across studies, they are more relevant to the population under study. Therefore, this score constitutes a tool for public health that can be useful in measuring the extent to which Costa Rican adolescents adhere to healthy diets. However, TCRAD score should not be static. As new scientific evidence emerges, especially when collected in the target population, the TCRAD score should be modified to reflect the dietary components with the best evidence of association with chronic disease, as has been done for diet quality indexes widely used internationally (90, 91).

The strengths of this study include the development of a new score to assess adolescent dietary patterns within their cultural context and dietary habits. This approach is useful to assess the cumulative effect of the overall diet on disease outcomes as pertinent to the population being studied. The use of dietary patterns avoids focusing on single foods or nutrients, and instead assesses combinations of food that may have additive or synergistic effects on disease (62). Furthermore, we used food records, which is an accurate and acceptable gold standard (62, 92) to validly assess diet of adolescents and create the TCRAD score.

While the TCRAD score showed the expected correlations with foods and nutrients of interest, suggesting internal validity, correlations were mostly of weak or moderate strength. Further, while we followed definitions of previously used traditional diet quality score, it is feasible that using alternate food and nutrient components and/or scoring may produce slightly different results. In addition, fewer boys than girls participated in the study, which could skew results since girls tend to have healthier dietary habits than boys (37, 41–43). Nonetheless, all analyses were adjusted for sex to lessen this bias. Despite these limitations, the Traditional Costa Rican Adolescents Diet score is a helpful tool to capture the diet quality of Costa Rican adolescents in a valid and culturally appropriate manner. The score can help detect subgroups of adolescents at higher risk of consuming diets with low TCRAD score and this information could be used to influence public nutrition policies and programs for non-communicable disease prevention. A high- traditional Costa Rican Adolescents score developed during adolescence may translate into a lower risk of obesity, diabetes, and CVD in adulthood (93).

## Data Availability Statement

The raw data supporting the conclusions of this article will be made available by the authors, without undue reservation.

## Ethics Statement

This study was approved by the Bioethics Committee of the Costa Rican Institute for Research and Education in Nutrition and Health (INCIENSA). The study protocol was approved under number IC-2007-01. All adolescents who participated in the study gave their informed assent verbally and wrote on the informed assent form. Likewise, the adolescents required to have the informed consent form signed by their parents to participate in the study. All guidelines for human subject research were strictly followed, in accordance with the international regulations, and specifically with Law 9234 Regulatory Law of Biomedical Research, which regulates biomedical research in Costa Rica.

## Author Contributions

RM-R: conceived and designed the study, collected, analyzed, and interpreted the data, and wrote the manuscript. JO'N and ML-B: contributed importantly to the analysis and interpretation of data and assisted in writing the manuscript. JM: made central contributions in the analysis and interpretation of data and assisted in writing the manuscript. All authors read and approved the final manuscript.

## Conflict of Interest

The authors declare that the research was conducted in the absence of any commercial or financial relationships that could be construed as a potential conflict of interest.

## Publisher's Note

All claims expressed in this article are solely those of the authors and do not necessarily represent those of their affiliated organizations, or those of the publisher, the editors and the reviewers. Any product that may be evaluated in this article, or claim that may be made by its manufacturer, is not guaranteed or endorsed by the publisher.
