# Supplementary figures and images for: A Traditional Costa Rican Adolescents' Diet Score Is a Valid Tool to Capture Diet Quality and Identify Sociodemographic Groups With Suboptimal Diet
Source: Front Public Health. 2021 Aug 12;9:708956. doi: 10.3389/fpubh.2021.708956 (PMC8397381; doi:10.3389/fpubh.2021.708956)

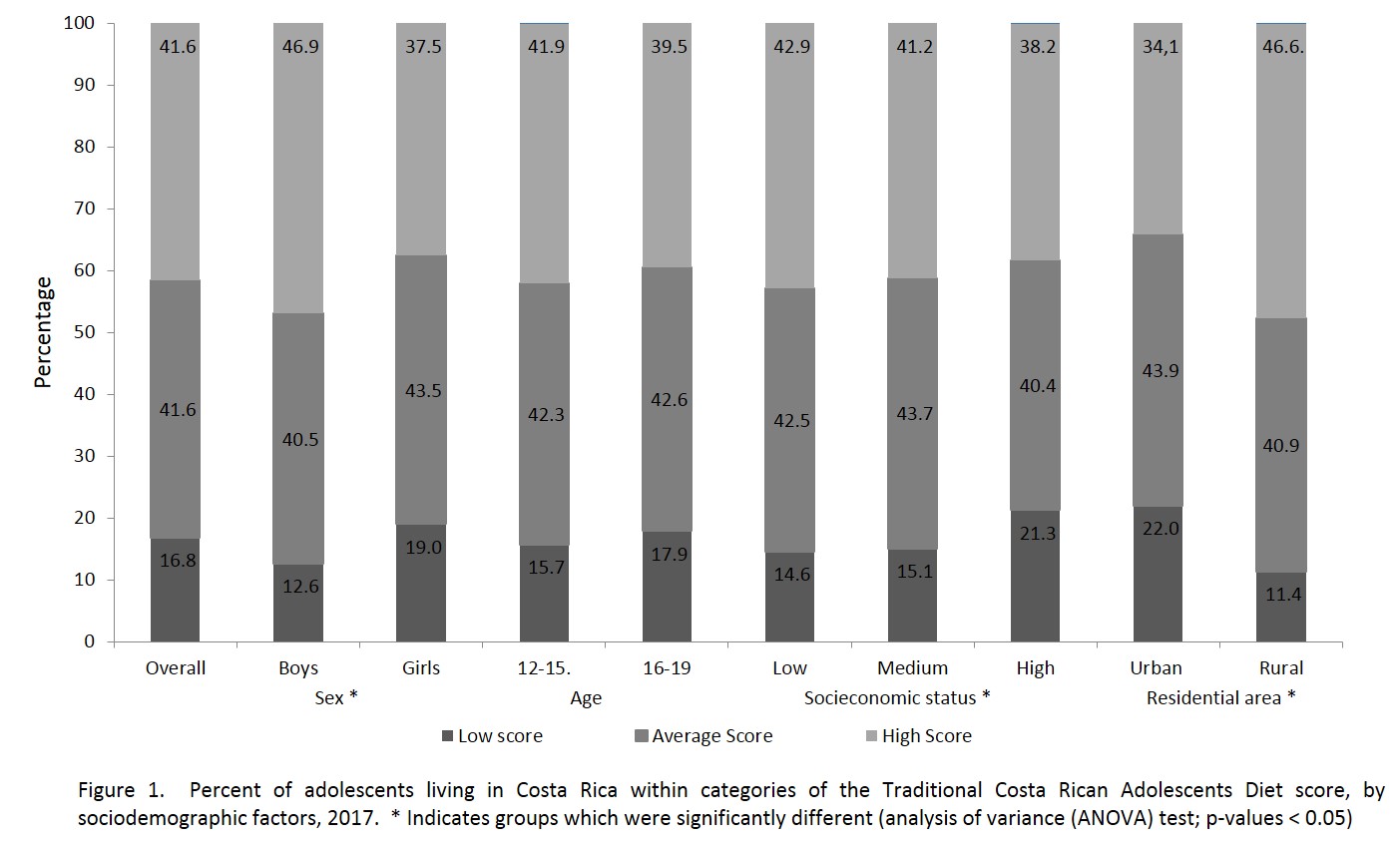

Supplement: Supplementary file 1 [file Image_1.JPEG]
